# Editorial: Fertility preservation in the pediatric population

**DOI:** 10.3389/fendo.2023.1149532

**Published:** 2023-02-16

**Authors:** Asma J. Chattha, Mahmoud Salama, Yasmin Jayasinghe

**Affiliations:** ^1^Department of Pediatric and Adolescent Medicine, Mayo Clinic, Rochester, MN, United States; ^2^Department of Obstetrics, Gynaecology and Reproductive Biology, Michigan State University, East Lansing, MI, United States; ^3^Department of Obstetrics and Gynaecology, University of Melbourne, Royal Women's Hospital, Parkville, VIC, Australia; ^4^Oncofertility Program, Royal Children's Hospital, Melbourne, VIC, Australia; ^5^Murdoch Children's Research Institute, Melbourne, VIC, Australia

**Keywords:** pediatric, adolescent, oncofertility, fertility preservation, ovarian tissue cryopreservation, testicular tissue cryopreservation, transgender, disorders of sexual differentiation (DSD)

The Frontiers in Endocrinology Research Topic on fertility preservation in children invited authors from across the globe to participate in the dissemination of knowledge and awareness regarding the best fertility preservation principles in the pediatric population. Although long considered a problem only in adults and post pubertal individuals undergoing cancer therapy, assisted reproductive technologies have rapidly advanced to include ovarian and testicular tissue preservation. This now allows prepubertal patients and families who were previously excluded from fertility conversations, to be included in these profoundly important discussions, which may provide hope for future attempts at parenthood (1). Fertility preservation is now considered for any medical condition requiring gonadotoxic treatment with curative intent, as well as those causing premature gonadal decline. This means that oncofertility care is now being rapidly expanded to include children with non-oncologic conditions affecting fertility such as genetic, rheumatologic, nephrologic disease, and hematologic conditions requiring bone marrow transplant, as well as the transgender population (1). However, many knowledge gaps exist in the pediatric population, which this Research Topic sought to address.

Disparities in oncofertility care across the globe are well described, both in high and low resource settings (2, 3). Many centers lack best practice oncofertility guidelines for children facing fertility-threatening diagnoses and treatment plans, resulting in significant distress for survivors (4). Furthermore, different aspects of oncofertility care are in different stages of translation. Ovarian tissue preservation has transitioned into standard practice, but requires ongoing monitoring in the young, while testicular tissue preservation is still experimental in humans (5, 6).

In this Research Topic, authors were invited to present their research on optimal methods, timing, and outcomes on fertility preservation in children and adolescents. Data on new populations eligible for fertility preservation is highlighted in this Research Topic.

Barrett et al. describe successful oocyte cryopreservation in 19 out of 20 transmen aged 12–20 years (median age 17 years).This is an important study since much of the previously published data is derived from the adult population. Two participants had been on testosterone, which was discontinued during oocyte collection. Around two thirds of patients cryopreserved at least 10 mature oocytes with many patients additionally freezing immature oocytes. There was no difference in outcome in those who attempted oocyte cryopreservation who had been on oral contraception, puberty blockers, or testosterone, compared with those who were naïve to hormonal therapy. Importantly, the use of dysphoria protection protocols in accordance with the World Professional Association for Transgender Health were implemented (7). This included using appropriate language and pronouns, avoiding triggering terminology, avoiding pelvic examination, and utilizing transabdominal ultrasound monitoring in the majority of cases. Similar to other studies on children, this study highlighted the high desire for family building in the gender diverse population and the high uptake of fertility preservation when barriers are reduced and culturally sensitive care is provided (8).

Highlighting the need to expand fertility preservation to conditions outside cancer therapy, two very important articles focus primarily on fertility preservation practices in pediatric patients with hemoglobinopathies and disorders of sexual development (DSD) (Bedrick et al.; Siebert et al.). The care of patients with DSD requires a multidisciplinary expert approach. Pitfalls in diagnosis after donor transplant are highlighted in this Research Topic by Li et. al. (9), who describe the importance of a detailed history and examination in patients presenting with premature ovarian insufficiency and XY karyotype (due to bone marrow transplant from a male sibling donor). Without exploration of this history patients could be misdiagnosed with Swyer syndrome instead of chemotherapy induced premature ovarian insufficiency. These are important clinical lessons for pediatric and adolescent oncology, endocrinology and gynecology clinicians alike, when puberty is delayed after cancer treatment.

There is intense interest in innovative fertility preservation techniques, including fertoprotective agents, which may be used as gonadal protectants during chemotherapy. In this Research Topic, Feng et al. explored if co-administration with melatonin, a free radical scavenger and a broad spectrum antioxidant, could reduce cyclophosphamide-induced primordial follicle loss in mice. The authors demonstrated that co-treatment with melatonin significantly prevented cyclophosphamide-induced apoptosis, of ovarian granulosa cells through inhibition of mitochondrial apoptosis pathways. Anti-mullerian hormone (AMH) expression was maintained, preventing non-growing follicle activation, maintaining ovarian reserve, and increasing litter size, providing new evidence for melatonin as a potential adjuvant chemotherapy agent of the future.

With respect to advances in gonadal tissue preservation, Moussaoiu et al. (10) reported the feasibility and safety of testicular tissue preservation from a Swiss multi-center network, adding to the currently limited body of literature on this technology (**Figure 1**). This study demonstrated high acceptance rates of testicular tissue preservation by families (90%), despite the experimental nature of the procedure. Importantly, the authors evaluated the quality of gonadal biopsies in a population of whom approximately 50% had received prior moderate gonadotoxic risk therapy (median cyclophosphamide equivalent dose of 5.5 mg/m^2^). This has not previously been well reported. Approximately 30% of the study population had a diagnosis of leukemia, where common consensus is to offer fertility preservation as an interval procedure (prior to bone marrow transplant) when minimum residual disease is negative (11).Tumor cells were found in one biopsy (through immunohistochemistry), highlighting the importance of pathological evaluation of all samples and the need for advancements in molecular technologies to detect malignant cells in gonadal tissue prior to transplantation.

**Figure 1:**
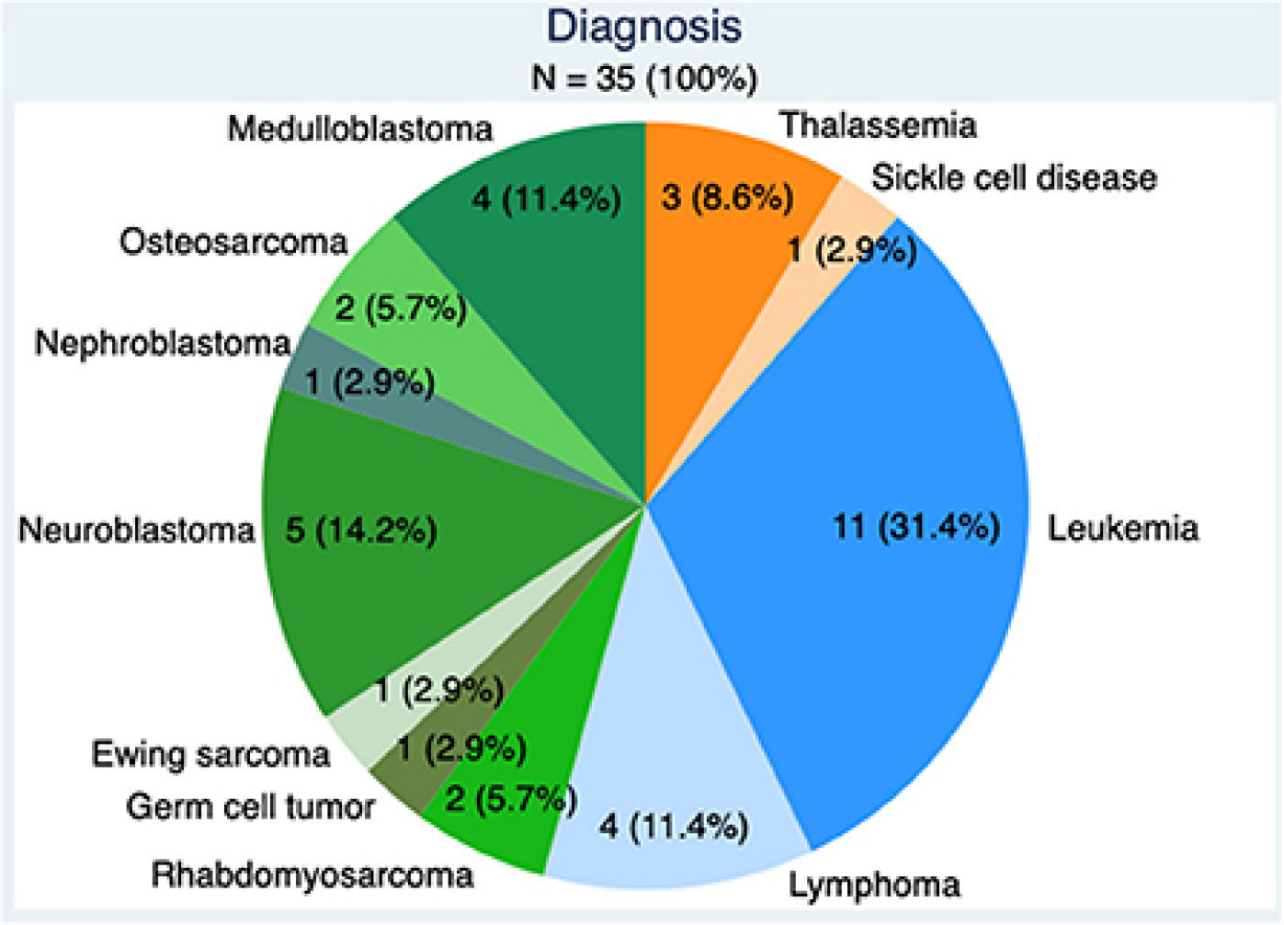
Underlying diagnoses requiring gonadotoxic therapy in boys: benign conditions (orange) Hematologic malignancies (blue) and solid tumors (green).

An improved understanding of the reproductive capability of collected gonadal tissue was further discussed by Baston-Bust et al., who studied a German cryobank focusing on ovarian tissue cryopreservation. The authors suggested that examination for follicle density be undertaken in order to plan the number of cortex pieces to transplant in the future when parenthood is required. The results of both of these studies highlight the knowledge gaps in pediatric oncofertility, and the importance of oncofertility registries in monitoring efficacy of fertility preservation technologies in the future (12). Further highlights reported on the successful achievements of the Fertiprotekt network of 150 centers, founded in 2006, which have now undertaken over 300 ovarian tissue preservation procedures in German-speaking countries, and counselled 60 patients aged 15 years or less, demonstrating the importance of achieving equitable care and meaningful research outcomes through sustained collaboration.

Finally, a novel study on the potential to harness the power of social media to disseminate knowledge on the impact of cancer treatment on fertility and fertility preservation options explores questions on how best to communicate with digital savvy adolescents and young adults in ways they may find more familiar. (Martinez-Ibarra et al.).

## Author contributions

All authors contributed to conception, critically evaluating the drafts and approving the final draft.
